# Teacher–student relationship at university: an important yet under-researched field

**DOI:** 10.1080/03054985.2014.921613

**Published:** 2014-05-21

**Authors:** Gerda Hagenauer, Simone E. Volet

**Affiliations:** ^a^ Salzburg University, Austria; ^b^ Murdoch University, Perth, Western Australia

**Keywords:** *teacher–student relationship*, *higher education*, *faculty–student interaction*

## Abstract

This article reviews the extant research on the relationship between students and teachers in higher education across three main areas: the quality of this relationship, its consequences and its antecedents. The weaknesses and gaps in prior research are highlighted and the importance of addressing the multi-dimensional and context-bound nature of teacher–student relationships is proposed. A possible agenda for future research is outlined.

## Introduction

In 1995, Baumeister and Leary published a review article focusing on the human need to belong, proposing the ‘belongingness hypothesis’, that ‘human beings are fundamentally and pervasively motivated by a need to belong, that is, by a strong desire to form and maintain enduring interpersonal attachments’ (p. 522). Subsequent research has demonstrated that quality relationships have an impact on human beings with respect to motivation, social competence and wellbeing in general (e.g., Bergin & Bergin, 2009), but also in regard to specific outcomes across different educational contexts.

In this article, we focus on the higher education or university context, and on one particularly significant relationship within that setting: the teacher–student relationship (TSR). The significance of the interpersonal relationship between students and teachers for students’ successful school adjustment has been widely recognised in research addressing kindergarten, primary and secondary education (Bernstein-Yamashiro & Noam, 2013; Roorda, Koomen, Spilt, & Oort, 2011). The awareness of the importance of this relationship for schoolteachers has also steadily increased, although this aspect has been much less frequently explored than the association between teacher–student relationships (henceforth, TSR) and students’ learning (Spilt, Koomen, & Thijs, 2011). However, while investigations of TSR at school have predominantly focused on well-established research traditions of self-determination theory (SDT) (Deci & Ryan, 2002) and attachment theory (AT) (Cassady & Shaver, 2008), and results from the significant body of research on the social factors of student motivation (Juvonen, 2006), TSR in higher education has been less comprehensively and less systematically examined by researchers. There are far fewer studies on TSR in higher education than in the school context. Furthermore, the limited studies of TSR in higher education often lack a clear theoretical/conceptual framework.

We argue that the investigation of TSR should be extended, as it is important for higher-education research for at least three reasons:

First, many universities worldwide have relatively large student drop-out rates, with high human and financial costs (for the USA, for instance, see Schneider & Yin, 2011). Investigation of TSR is relevant if enhancing TSR can help to reduce this negative trend.

Secondly, the need to belong also affects university teachers. Thus, it is likely that a positive ‘relational classroom environment’, including positive interactions and relationships, may also have positive effects on the teachers themselves (e.g., on teachers’ positive emotions; see Hagenauer & Volet, 2014), as relational approaches to teaching suggest (e.g., Graham, West, & Schaller, 1992; Wilson, 1992).

Thirdly, given the increasing importance ascribed to excellence in university teaching as part of the discourse on ‘Scholarship in Teaching and Learning’ (e.g., Kreber & Cranton, 2000; Trigwell & Shale, 2004), the significance of TSR requires detailed investigation. For example, the quality, establishment, and effects of social factors such as TSR should be explored in greater depth, given their likelihood as preconditions of excellence in teaching and learning at university. National studies such as the National Student Survey (GB), the National Survey of Student Engagement (USA), and the Course Experience Survey (AUS) have broadly examined different aspects of excellence in teaching. However, they address aspects of TSR only indirectly and superficially, such as asking about academic support (e.g., ‘I have been able to contact staff when I needed to’; item from the National Student Survey; HEFCE, 2011). Thus, we conclude that research on TSR in higher education should be an integral part of the larger body of research and discourse on the quality of teaching and learning in higher education.

The aim of this article is to analyse critically previous research on TSR in higher education and to identify several areas in which empirical evidence is limited. Prior investigations of the concept of TSR have originated from various research traditions, including educational and psychological theories and communication research. This review focuses exclusively on research from an educational or psychological perspective.

Following a brief description of the literature search methodology, the article is organised in four parts. First, we address the quality of TSR in higher education. Second, we examine studies that have explored the consequences of TSR, focusing on the effect of TSR on students, as teacher effect is almost absent from empirical research. Third, we discuss empirical work focusing on the development of TSR and describe how interactions, their frequency and quality may contribute to that process. Fourth, we present a heuristic framework that brings together the aforementioned, and propose an agenda for future research on TSR.

## Methodology

The selection of relevant literature consisted of two phases. First, a systematic search was undertaken through selected databases in education, psychology and social science (ERIC, Psyndex, Psych Info). Second, a snowball procedure (involving follow-ups on some of the references cited by the studies identified in the initial search) was applied. The inclusion criteria used in this two-phase approach were that papers had to: (1) be empirical in nature (quantitative or qualitative); (2) deal either with ‘teacher–student interaction’ (staff-student interaction; faculty-student-interaction) or ‘teacher–student relationship’ (since these terms were frequently used interchangeably); (3) focus on students’ or teachers’ perspectives or both; and (4) be published between 1997 and 2012 (a range of 15 years); occasional references to earlier publications were permitted.

## The quality of TSR

In this section, we discuss the conceptual and operational problems associated with the concept of TSR in higher education. This is followed by an examination of the multi-dimensional and context-dependent nature of TSR. We then review empirical studies that have addressed various aspects of the nature of TSR.

### Conceptualisation and operationalisation difficulties

Conceptualising TSR in higher education is not easy, as the field is under-explored and multifarious. Inconsistencies in the available literature are due to the typical lack of clearly defined conceptual/theoretical frameworks (such as SDT or AT, as applied in research on TSR in schools) on which the studies can be based. This has resulted in several empirical studies that have operationalised TSR in higher education very differently, making it difficult to analyse them as a unified group and draw comparisons.

From our perspective, the lack of a coherent conceptualisation of the nature/quality of TSR can be attributed to three main factors:

First, most of the studies do not treat TSR as the ‘variable-of-interest’ or (from a quantitative methodological view) as the ‘dependent’ variable; rather, they use it as an explanatory or ‘independent’ variable amongst others to explain students’ outcomes (e.g., student motivation, drop-outs). This has led to the development of well-defined conceptual frameworks for outcomes such as student drop-outs (Tinto, 1975), but no comprehensive conceptual frameworks have been developed for the various ‘explaining’ variables like TSR.

Secondly, the few studies that have *de facto* focused on TSR as the variable-of-interest are primarily qualitative. They provide fruitful insights into, for instance, teacher and student perspectives on positively or negatively experienced TSR (e.g., Anderson & Carta-Falsa, 2002), but do not take these empirical findings to the next level, namely the derivation of broader, more generalisable dimensions of TSR.

Thirdly, in investigating TSR, much of the literature focuses on teacher-student (or faculty-student) interactions, without describing the quality of TSR. In several studies, the frequency of interactions was the main focus of investigation (for an overview, see Lamport, 1993). Generally, investigations of the frequency of teacher–student interactions show that the more often students have out-of-classroom interactions (e.g., office visits) with their university teachers, the better the quality of the relationship and the more connected the students to the university. However, as Dobransky and Frymier (2004) and Komarraju, Musulkin, & Bhattacharya (2010) maintain, the frequency of interactions does not enable conclusions about the quality of these interactions or the quality of the underlying relationship. Not all instances of interactions with university teachers are necessarily positive in nature, and thus do not automatically lead to positive outcomes. Furthermore, as Baumeister and Leary (1995) argued, interactions must be distinguished from relationships. Although some more recent empirical studies have made valuable attempts to assess the quality of teacher–student interactions (e.g., Frankel & Swanson, 2002), findings remain of limited value in terms of knowledge about the quality of TSR, as situation-bound interactions should be regarded as the antecedents of TSR, not its constituents.

### Accounting for the multi-dimensionality and context-dependency of TSR

Despite the aforementioned difficulties in comparing research findings, studies on TSR in higher education provide some initial insights into its quality. From the school research, it is clear that TSR cannot be conceptualised as a one-dimensional construct; rather, it is multi-dimensional in nature.

A number of researchers have developed instruments and scales to assess the quality of TSR in schools, most of them grounded in AT or SDT. AT is a theory based on attachments or relationships; in contrast, in SDT, relationships are not the only factor at work, as the sense of relatedness is regarded as one of three basic psychological needs that influence human motivation.

Overall, AT has provided the most differentiated instruments regarding the quality of TSR. One internationally recognised instrument used to assess TSR is the Teacher–student-Relationship Scale (Pianta, 2001), which distinguishes between the TSR dimensions of closeness, conflict, and dependency. SDT researchers who focus primarily on the relatedness need, also tend to apply a multi-dimensional approach to TSR and frequently refer to AT when putting the TSR construct into operation (e.g., Ryan, Stiller, & Lynch, 1994). However, conceptualisations and derived operationalisations of the TSR construct differ substantially between studies. The lack of consistency in conceptualisations of TSR in school research limits the potential use of existing instruments validated in school contexts in the higher-education context.

Two additional factors prevent the mere application of concepts from school-related TSR research to the higher-education context. One factor relates to the characteristics of the interactants and the educational environment, which have a direct impact on the quality or nature of TSR.

For example, in higher education, TSRs are formed between adults, whereas in the school context, relationships are formed between an adult and a child or an adolescent, discussed later in this article in relation to the ‘care construct’. Moreover, teaching settings tend to be more fragmented at university, with less frequent interactions between teachers and students. In addition, teaching is just one scholarly activity expected of university educators, with quality research typically receiving greater recognition than quality teaching in the academic community.

The second factor concerns specific dimensions related to TSR that either diminish or escalate in importance over time. For instance, the dimension of ‘dependency’ in TSR applies to research on younger students (e.g., kindergarten and primary school), but loses importance as students age and become more independent learners. Consequently, researchers in secondary education have eliminated the dimension of dependency in their operationalisations of TSR (Ang, 2005).

**Figure 1.**
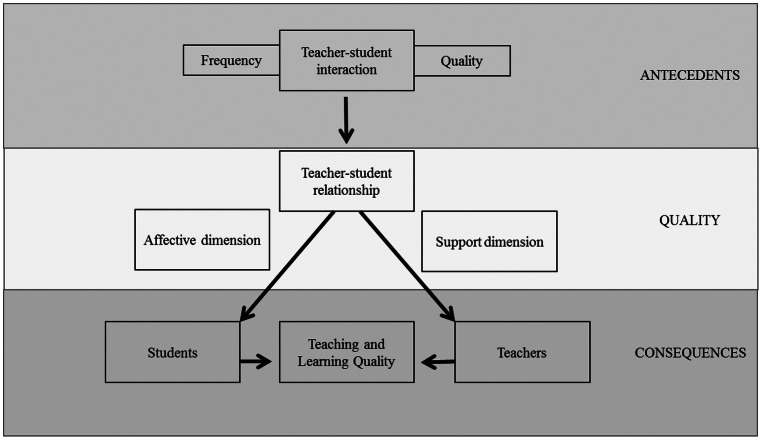
Exploring TSR in higher education—a heuristic framework for future research

In examining the higher-education literature that has addressed TSR, and drawing on findings of school studies, we tentatively identified two main dimensions that can be differentiated when describing TSR in higher education (see Figure 1, middle section on Quality):

1. The *affective dimension*, which describes the bond built between students and teachers, forming the basis for secure and affective positively experienced relationships.
2. The *support dimension*, which describes the support that must be provided through TSR for students’ success at university (e.g., teachers setting clear expectations, answering emails promptly).

In the following paragraphs, we detail empirical studies from which this distinction has been drawn. Beforehand, we direct the reader’s attention to another important characteristic of TSR: its context-dependency. In addition to its multi-dimensional nature, we also maintain that TSR must be regarded as a context-dependent construct, as previous empirical findings have revealed.

For instance, Hsieh (2012) demonstrates that Chinese teachers have a different understanding of TSR from British teachers. Also, students studying outside their home country often bring different expectations to TSR (Zhou, Jindal-Snape, Topping, & Todman, 2008). Furthermore, the subject being studied affects students’ perceptions of TSR, shown by Sander, Stevenson, King, & Coates’s (2000) findings in which psychology students rated the importance of personal relationships with teachers higher than business students did. Results from a team of Finnish and Australian researchers suggest that this is also the case for teachers (e.g., the teaching approach, including relational issues, of teachers in the ‘soft sciences’ differs from that of ‘hard science’ teachers; Lindblom-Ylänne, Trigwell, Nevgi, & Ashwin, 2006; Parpala, Lindblom-Ylänne, Komulainen, Litmanen, & Hirsto, 2010). (Further examples of the context-dependency of TSR abound, but are beyond the scope of this article.)

TSR varies not only between contexts but also within an actor in different contexts. For instance, Lindblom-Ylänne et al. (2006) found that teaching practices of the same university teachers differed, dependent on course format (seminars/lectures). The teaching context not only influences the teaching approach, but also affects TSR, insofar as opportunities to approach students and to build relationships with them within formal teaching-and-learning settings are much greater in seminars (workshops/tutorials) than in lectures. This context-dependency makes the formulation of an overarching definition of TSR rather difficult. Similarly vexing is the question of what characterizes or defines good teaching at the university level, an issue that—according to Martens and Prosser (1998) and Trigwell (2001)—remains open to debate.

### Empirical findings on the quality of TSR

In this section, we review a number of empirical studies that have addressed the two aforementioned broad dimensions of TSR in higher education: the affective and support dimensions.

*Affective dimension.* Many different aspects of TSR are comprised in the affective dimension (e.g., honesty, trust, respect). One of these factors is ‘care for students’ which will be outlined in the following section as it is heterogeneously discussed in the higher education context. There is strong empirical support in the general literature for the idea that ‘caring’ for students is regarded as a humanistic value. The unquestionable moral responsibility for schoolteachers to care for their students has been discussed from a normative perspective by Noddings (1995) and Goldstein (1999). It has also been further empirically validated by Meyer’s studies (2009) on student teachers, as well as by Oplatka’s (2007) research on primary and secondary school teachers. Gholami and Tirri (2012) attempted to assess empirically the multi-dimensional construct of ‘care’ in school teaching. One of the subcomponents of their instrument relates to nurturing ‘students’ character’ and applying ‘respectful didactics’.

The relevance of ‘caring behaviour’ within TSR in higher education and the boundaries of TSR in that context have received less attention in the literature. As mentioned earlier, whereas TSR in school is formed between a child or an adolescent and an adult, TSR at university is characterized as an adult–adult relationship (see Halx, 2010, on the issue of considering undergraduate students as adults). There are different expectations for this adult–adult relationship, mainly regarding the degree of expected dependency or independency. The dependency of younger learners on their teacher is much higher in school, an accepted feature of the relationship, fostering teachers’ urges to ‘care for’ or ‘take care of’ the still dependent learner. In the university context, independent (adult-like) behaviour is expected from students (e.g., self-organisation, independent studying). This expectation raises questions, such as: Given the assumption of independence, do university teachers have an obligation to display caring behaviour? If so, how is ‘care’ defined in this adult–adult teaching and learning context?

Empirical research findings illustrate how this question has been considered from different perspectives with various results. While Fitzmaurice’s (2008) findings show university lecturers viewed care as being important, Lähteenoja and Pirttilä-Backman’s (2005) study reveals differing opinions among a sample of Finnish university lecturers asked their opinions on the importance of connecting with first-year students, and explicitly attempting to integrate them into the department. Explicit attempts to promote student integration can be regarded as a specific form of ‘caring’. Although it was termed ‘student integration’ in the Finnish study, the strategies lecturers reported showed high overlap with the ‘care concept’. While some agreed that student integration was ‘beneficial for both teachers and students’, others regarded it as ‘unnecessary’ and even ‘harmful’, reporting that they did not want to coddle students, and that students should study independently without extra care from staff. Others felt caring for students at university important, believing that a safe environment should be created, with positive opportunities for interaction between students and teachers, and among students. Overall, the study reveals disagreement over the importance of caring relationships at university, which may be due to differing or unclear understandings of the ‘care concept’ among faculty interviewed.

Other literature addresses the importance of the care concept for factors involved in student learning, such as intrinsic motivation (Komarraju, Musulkin, & Bhattacharya, 2010). In Komarraju et al.’s study, factors such as respect and connectedness (as well as care) were regarded as important features of a positive TSR. These sub-components of TSR mirror the affective-based understanding of TSR outlined above.

*Support dimension.* Fitzmaurice’s findings (2008) match Komarraju et al.’s (2010), and supplement the description of the quality of TSR with the support dimension: Irish university lecturers characterised a good relationship with students, using adjectives such as honest, respectful, trustworthy, safe, fair, encouraging, caring and supportive. Similarly, university lecturers in Anderson and Carta-Falsa’s (2002) study described a positive interpersonal TSR at university as open, respectful, supportive, comfortable, safe and enjoyable (in order of importance; see also Jacklin & Le Riche, 2009).

However, although these findings indicate that establishing some sort of connection (reliant on mutual respect, fairness, safety etc.) is considered important for TSR, they also show that the interpersonal relationship between students and university teachers is considered one that must be ‘balanced’. This raises the question: How close should TSR in higher education be, and when does it become ‘too close’?

The need for students to remain within the bounds of a professional (working) relationship is empirically described in Holmes, Rupert, Ross & Shapera’s (1999) study, in which students were asked to rate the appropriateness of various teacher behaviours. The results show that students perceived behaviour that went beyond academic roles as inappropriate for TSR, with items connected to the ‘friendship relationship’ dimension particularly, rated low in terms of appropriateness by the students (e.g., the teacher attends a student’s party; goes shopping with a student). Sibii (2010) described the role of the teacher in TSR as ‘a friendly individual but not a friend’ (p. 531). Furthermore, Holmes et al. (1999) discussed the risk of overly close and informal relationships: allowing relationships to become too close or informal could be risky for university teachers and students due to their hierarchical nature and unequal power distribution. In particular, cross-gender TSR could be viewed as crossing the line if interactants became too close. Students in Holmes et al.’s (1999) study rated ‘sexual relationships’ in the higher-education context as highly inappropriate (e.g., the teacher and student date; the teacher tells a student that he/she is attracted to him/her). In conclusion, these findings suggest that TSR in higher education, particularly regarding ‘closeness’, can be perceived as a balancing act in which both teachers and students must be mindful of boundaries, and TSR not be overly amicable or informal.

A balancing act is required also in terms of supportive or helpful behaviour—the second sub-dimension discussed in the previous section. Research shows that students need support from their teachers (e.g., clear expectations; Åkerlind & Jenkins, 1998) to be successful in class, yet Devlin and O’Shea’s (2012) study showed that students wanted to be challenged by teachers. From students’ perspectives, teachers who set high (academic) expectations and who did not overly ‘nurture’, ‘spoon-feed’, or ‘coddle’ students were rated most positively.

Another concept frequently discussed within the TSR-framework in higher education is the *approachability* of teachers. Approachability is difficult to assign to either the affective or the supportive dimension, as approachability itself can be regarded as a multi-dimensional construct requiring conceptual clarification.

The multi-dimensionality of the concept of approachability is illustrated in Denzine and Pulos’s (2000) study, in individual items the authors assigned to the ‘approachable’ and ‘unapproachable’ categories of teacher behaviour based on student views. Highly approachable teachers were characterised by behaviour such as knowing students’ names, staying in class to meet students, saying ‘hi’ to students on campus, smiling often, and exhibiting warm and caring behaviour. Unapproachable behaviour was described by items such as ‘talks down to students’, ‘misses office hours’ and ‘appears bored when teaching’. It is noteworthy that the putting into operation of the construct of approachability by Denzine and Pulos (2000) shows a high degree of overlap with the concept of ‘teacher immediacy’ in Communication Research (Sibii, 2010). This confirms our contention that approachability as a construct remains open to definition.

Denzine and Pulos (2000) argued that teacher approachability is an important quality that must be guaranteed in order to facilitate positive teacher–student interactions. Stephen *et al.* (2008), in a British higher-education study, support this, showing that the approachability of lecturers is relevant not only for TSR, but also for an overall feeling of connectedness to the university and preventing students from becoming alienated from the university. Teacher approachability also features in an Australian higher-education study, showing the significance of approachable and available university lecturers for the adaptation process of first-year students from a low socio-economic background (Devlin & O’Shea, 2012). Approachable lecturers and tutors who answered students’ questions promptly, and clearly communicated expectations with regard to assignments, were described as very helpful for students’ success in learning and adjusting to university.

Based on the empirical studies discussed above, we can conclude that approachability consists of two dimensions, a support and an affective dimension; however, alone it does not appear to represent a separate dimension of TSR. Rather, approachability can be regarded as one characteristic amongst many, frequently used interchangeably and without a clear definition in connection to TSR in higher education. This leads us to conclude that teachers are described as approachable when they support students in their study progress (e.g., if they are available for help-seeking students, etc.), which seems to justify the attribution of a support dimension to TSR. Additionally, teachers in the studies cited above were also perceived as approachable by their students when the latter felt that their teachers could be trusted and listened to their problems (academic and/or personal). From our perspective, this addresses the affective dimension.

In summary, previous research suggests that TSR can be described using a range of concepts including closeness, care, connection, safety, trust, honesty, fairness, respect, openness, support, encouragement, availability and approachability. Although these concepts have largely been explored without a clear theoretical underpinning and have often been derived directly from the empirical material itself, they nevertheless suggest that TSR in higher education, as in schools, should be conceptualized as a multi-dimensional construct. We have made a first attempt to integrate these constructs into tentatively super-ordinate dimensions, differentiating between affective and support dimensions. However, further studies are needed to refine the conceptualisation of the quality of TSR in higher education on a solid empirical basis.

## The consequences of TSR on students and teachers

Based on the ‘belongingness hypothesis’ (Baumeister & Leary, 1995), it is expected that TSR influences both groups of actors involved in the relationship: students and teachers. Despite some empirical evidence that TSR is crucial for students’ successful learning at university (see the following chapter), the association between TSR and teacher factors is under-researched across all sectors of education, from school to university, highlighted by Spilt, Koomen, & Thijs’s (2011) review of this literature. The research gap is particularly striking in higher education (Komarraju et al., 2010; Wilcox, Winn, & Fyvie-Gauld, 2005), which makes it difficult to generate valid expectations about the possible consequences of TSR for university teachers. One Finnish study (Lahtinen, 2008) found university teachers experienced negative emotions in interactions with their students, though drew no conclusions regarding the possible long-term effects of these negative experiences for teachers. Therefore, as a result of limited research on the effects of TSR on university teachers, this review concentrates on work that examined the effects of TSR on university students, and concludes by calling for increased attention to studying the consequences of TSR on university teachers in future research.

### TSR and university students

A significant body of research focusing on the importance of various factors (including TSR) on students’ successful study progress can be found in the higher-education literature from recent decades. TSR can be regarded as a precondition of successful learning for all students, but seems to be of particular relevance for at-risk students in terms of study retention or drop-out decisions. These studies on TSR in higher education have largely arisen from the need to explain and prevent the phenomenon of student drop-out. Drawing heavily on Tinto’s framework (1975) of student drop-out, researchers have addressed the quality of academic and social integration as influential in determining whether students stay at or leave university (e.g., Oseguera & Rhee, 2009; Pascarella & Terenzini, 2005).

Research by Palmer, O’Cane, & Owens (2009) shows that the likelihood of remaining at university was higher for students who developed a sense of belonging to the university, as their study satisfaction was increased through connectedness. Development of a feeling of belonging is of particular importance in the first year of study, as most decisions to drop out are made during this year (Christie, Munro, & Fisher, 2004). Furthermore, many first-year students enter university with unclear expectations and relatively high levels of uncertainty and anxiety, as studies focusing on experiences of first-year students have revealed (e.g., Gibney, Moore, Murphy, & O’Sullivan, 2011; Hazel, Tett, Cree, Hounsell, & McCu 2008). Brinkworth, McCann, Matthews, & Nordström (2009) found that Australian first-year students had unclear expectations not only regarding their role as students, but also regarding TSR at university. Over 80% of the sample expected to have ‘ready access’ to tutors and lecturers to facilitate successful study. Although they expressed awareness that studying at university is different from high-school learning, they expected similar conditions with regard to TSR. If students fail to connect to the university and their study subject for whatever reason (e.g., unclear expectations, as shown by Brinkworth et al., 2009), drop-out is often the result.

Although there is empirical support for the idea that peer relationships are the most important for students’ sense of belonging (Ramsay, Jones, & Barker, 2007; Strauss & Volkwein, 2004), relationships with teachers and tutors also play an important role in students’ decisions to complete their studies or to leave after the first year (Wilcox et al., 2005). Furthermore, positive relationships with university teachers not only contribute to the retention of students but also facilitate other factors, such as commitment (Strauss &Volkwein, 2004), effort (Lundberg & Schreiner, 2004), motivation (Rugutt & Chemosit, 2009; Zepke & Leach, 2010), satisfaction (Calvo *et al.*, 2010; Dobranska & Frymier 2004; Trigwell, 2005), engagement (Zepke & Leach, 2010), deep-learning approaches (Trigwell, 2005), achievement, and intellectual development (e.g., critical thinking, learning fundamental principles; Halawah, 2006). Pascarella and Terenzini (2005) empirically show the independent influence of TSR on students’ successful learning, controlling for various personal characteristics (e.g., gender, area of study, achievement orientation).

However, given the lack of longitudinal and experimental studies, the question of causality between TSR and students’ learning outcomes remains under-explored. One exception is an experimental study by Clark, Walker, & Keith (2002), but the experimental manipulation employed was very weak (i.e., one mandatory office visit should lead to desirable changes in students’ variables); as a result, these findings are inadequate for the extrapolation of more generalisable conclusions. Despite the lack of experimental and longitudinal studies, it appears that reciprocal influences between TSR and student factors can be assumed, as in the school context (Juvonen, 2006).

Along with the dearth of longitudinal studies, absence of comprehensive empirical research on TSR and its relevance for students’ learning must be addressed. Most of the reported studies originate from Anglo-American countries, with the majority of the research stemming from USA researchers. The dominance of USA-based research is also evident in school research. A meta-analysis (Roorda, Koomen, Spilt, & Oort, 2011) shows that of the 92 empirical papers dealing with TSR at school between 1990 and 2011, 77 were based on samples from the USA.

In summary, *longitudinal* and *experimental* studies on the association between student learning factors and TSR *across* different countries—relying on a comparable framework—are needed.

## The development of TSR based on interactions 

Studies of the development of TSR based on interactions are limited (Wilcox et al., 2005). Yet research in this area is vital to determine how the development of positive TSR can be fostered, with a view to achieving positive outcomes for both students and teachers and overall teaching quality. In this section, we discuss the importance of teacher–student interactions as antecedents of TSR.

Based on the assumption that TSR develops through ongoing interactions between students and teachers, the fundamental basis of TSR is the *occurrence* of these interactions. With regard to the beneficial effects of a positive TSR on students’ university adjustment, prior studies on the frequency of teacher–student interactions have painted a relatively alarming picture. Cotten and Wilson (2006) show that out-of-class interactions between students and their lecturers are infrequent and mainly task-focused. Few interactions go beyond course-related issues. This mirrors Jaasma and Koper’s (1999) findings showing that only 50% of an American student sample visited their teacher during office hours, and that these consultations were brief (mode = 6–10 minutes). Informal interactions before and after class were more frequent, but a third of the students reported that they had never contacted their teachers informally. This is consistent with Stephen, O’Connell & Hall’s results (2008): personal tutors at a British university reported many students did not approach them, even though their responsibility included both academic and social support. Students reported that they did not dare ‘steal the tutors’ time’, as they seemed ‘too busy’. The time factor appears to increase in significance as higher education develops towards mass education and time for personal contact with students becomes increasingly rare. This fits Jaasma and Koper’s (1999) findings showing that the perceived availability of time played a crucial role in determining whether students approached university staff or not. From the students’ perspective, (1) the uncertainty over whether lecturers were interested in forming relationships with them, (2) the impression that lecturers were under high time pressure, and (3) the lack of clarity regarding the benefits of interactions with university teachers were significant factors contributing to the infrequency of these interactions.

Furthermore, Jaasma and Koper’s (1999) study shows that students sometimes evaluated interactions as ‘costly’ if negative in nature (e.g., the rudeness of a lecturer responding to a question), or fear feeling compelled to exhibit desirable behaviour if the teacher knew them personally. Another factor shown to have an effect on the ease or difficulty of relationship building was ‘space’. When offices and study places (e.g., the library) were in separate buildings, and when lecturers did not have an office due to being part-time teachers departing immediately after lecturing, informal interactions were hampered (see also Cotten & Wilson, 2006).

With regard to TSR, research results on the frequency of interactions are important as they suggest that it might be difficult to establish positive relationships when interactions rarely occur. However, Braxton, Milem, & Sullivan’s (2000) study shows that positive relationships can be fostered not only through informal (out-of-class) interactions, but also in the more formal classroom setting through the use of active learning methods that support interactions in the classroom (e.g., discussions, group work). By means of active learning, the social integration of teachers and students is promoted in the classroom.

While the studies described above show that informal interactions between students and teachers do not frequently occur, such interactions do take place regularly in the formal context (e.g., in the seminar setting) and contribute to the development of TSR in that setting.

However, not only the occurrence of interactions is considered relevant for TSR, the quality of interactions also has a crucial role. To date, little is known about how interactions are perceived, evaluated and experienced by students and teachers. Therefore, future research should attempt to establish the importance of different kinds of interactions for TSR, as not every interaction between students and teachers is of equal quality and of equal relevance for the development of TSR. This is consistent with Docan-Morgan’s (2011, p. 20) proposal to ‘identify the turning point events’ in TSR.

## Discussion and conclusion

The overall aim of this paper was to provide an overview of research relating to TSR in higher education. TSR has emerged as an important construct in educational research within school and pre-school settings, but remains largely neglected in higher-education research. This review has shown that TSR should be regarded as a relevant construct in higher education as well, as it clearly affects students’ successful study progress, including factors such as course satisfaction, retention, learning approaches and achievement. It has also revealed that the empirical basis is less clear and comprehensive in terms of the consequences of TSR for university teachers. However, it is likely that TSR also affects university teachers, for example through their adoption of particular teaching practices, which in turn affects teaching quality.

We conclude that TSR should be regarded as a relevant research agenda for higher education. Several fruitful avenues for future research have been identified. These are presented in the following section as a heuristic framework, which may be useful in guiding future research endeavours on TSR in higher education.

## A heuristic framework and possible agenda for future research on TSR in higher education 

This review has identified three main areas for future research: one involves the conceptualisation and definition of the multifaceted construct of TSR, while the other two address the antecedents and consequences of TSR. Figure 1 depicts a heuristic framework for these possible emerging research agendas.

First, in order to develop a more profound understanding of TSR, one of the more urgent tasks is to increase our research efforts in terms of exploring the quality of TSR (see Figure 1; middle section on Quality). In this review, we emphasise the idea that TSR as a construct be regarded not only as multi-dimensional (currently distinguished by two subordinate dimensions, affective and supportive), but also as context-dependent. The complexity and context-dependency of the construct result in some conceptual and methodological challenges. From a conceptual perspective, the challenge remains to identify, strengthen, and define the sub-components of TSR in order to create a foundation for the development of more standardised operationalisations of TSR in higher education. At this point, methodological challenges emerge. The review of previous empirical work has shown that the majority of studies on TSR in higher education are primarily qualitative and explorative in nature. Furthermore, due to the lack of standardised instruments, the few studies that rely on quantitative methods are not reliably comparable. The context-dependency of TSR (e.g., with culture as an influencing factor) presents serious challenges for the development of standardised instruments and raises fundamental questions regarding the generalisability or contextualisation of such instruments (Volet & Summers, 2013), since what is being measured is by nature context-dependent. To advance research in this field, we suggest that future research validate the construct ‘TSR in higher education’ on a theoretical basis (= testing content validity, e.g., by establishing research cooperation among experts across different countries and also across different study subjects), as well as on an empirical basis (= testing construct validity, e.g., by applying confirmatory factor analysis across different groups, taking the context-dependency of TSR into account).

Secondly, we suggest extending understanding of the consequences of TSR for students and especially for university teachers. Furthermore, if TSR affects the quality of learning and teaching in higher education due to its effects on teachers and students, then it is an important agenda for research in higher education with direct implications for policy and practice (see Figure 1, bottom section on Consequences). This research agenda addresses the practical relevance of TSR. In terms of research methodology, multiple methods including quantitative and qualitative approaches will be required (e.g., Smith, 2006). For example, researchers might want to explore general associations between TSR and teachers’ teaching satisfaction by using self-report methods, such as questionnaires or interviews. Researchers may also take a situative perspective by questioning and examining associations between TSR and teaching practice. For such research, a combination of methods (for example, classroom observations combined with questionnaires or diaries) might be useful. Overall, the respective research question/focus will determine which method(s) will be the most appropriate and fruitful (see, for example, Gläser-Zikuda & Järvelä, 2008).

Thirdly, it is argued that a more comprehensive understanding of the development of TSR is imperative. In particular, the relevance of situative interactions and their evaluation in terms of quality by the interactants involved deserves further investigation (see Figure 1, top section on Antecedents). Longitudinal, developmental, and trait-variable-oriented perspectives call for approaches that will be capable of identifying a non-linear, interwoven and reciprocal relationship (e.g., by latent-growth modelling (LGM); Duncan, Duncan, & Strycker, 2006). From a situative perspective grounded in social practices, methods are required that will be capable of capturing real-life social interactions that have been identified as preconditions of TSR (Volet & Vauras, 2013).

In sum, we argue that TSR in higher education represents a relevant and promising area for future research in the field of higher education. Such research is expected to contribute to our understanding of the factors influencing the quality of learning and teaching in higher education (e.g., Kreber & Cranton, 2000; Trigwell & Shale, 2004). One of the greatest challenges will be to develop shared theoretical and conceptual understandings of TSR as a precondition for meaningful scientific communication, theorising and conceptualisation, as well as for study design. A second challenge will be to develop methods that can capture the complex, dynamic and context-specific phenomena under investigation. The heuristic framework outlined here represents an attempt to map possible future research agendas.

## Notes on contributors

Gerda Hagenauer is a Senior Scientist (Post-Doc) in Educational Sciences in the School of Education at Salzburg University, Austria. Her main research interest is in the area of quality of teaching and learning, focusing on emotional, social and cognitive factors while teaching and learning at school and university.

Simone Volet is Professor of Educational Psychology at Murdoch University, Perth, Australia. Her research interests are in the areas of the cognitive, metacognitive, motivational, emotional and social aspects of learning at university, teacher–students and student–student interactions, and issues related to cultural transitions in higher education.
